# New Equation for Predicting Pipe Friction Coefficients Using the Statistical Based Entropy Concepts

**DOI:** 10.3390/e23050611

**Published:** 2021-05-14

**Authors:** Yeon-Woong Choe, Sang-Bo Sim, Yeon-Moon Choo

**Affiliations:** Department of Civil and Environmental Engineering, Pusan National University, Busan 46241, Korea; ywchoe@pusan.ac.kr (Y.-W.C.); thfkthf@naver.com (S.-B.S.)

**Keywords:** entropy concept, friction factor, pipe flow, pipe friction coefficient

## Abstract

In general, this new equation is significant for designing and operating a pipeline to predict flow discharge. In order to predict the flow discharge, accurate determination of the flow loss due to pipe friction is very important. However, existing pipe friction coefficient equations have difficulties in obtaining key variables or those only applicable to pipes with specific conditions. Thus, this study develops a new equation for predicting pipe friction coefficients using statistically based entropy concepts, which are currently being used in various fields. The parameters in the proposed equation can be easily obtained and are easy to estimate. Existing formulas for calculating pipe friction coefficient requires the friction head loss and Reynolds number. Unlike existing formulas, the proposed equation only requires pipe specifications, entropy value and average velocity. The developed equation can predict the friction coefficient by using the well-known entropy, the mean velocity and the pipe specifications. The comparison results with the Nikuradse’s experimental data show that the R2 and RMSE values were 0.998 and 0.000366 in smooth pipe, and 0.979 to 0.994 or 0.000399 to 0.000436 in rough pipe, and the discrepancy ratio analysis results show that the accuracy of both results in smooth and rough pipes is very close to zero. The proposed equation will enable the easier estimation of flow rates.

## 1. Introduction

In order to accurately predict the flow discharges in pipe flow, not only the diameter and flow velocity, but also the discharge loss due to pipe friction, is very important. However, the most important factor in calculating flow loss in pipes is the friction coefficients. Existing equations are limited in accurately predicting friction factors due to material properties.

Studies with various experiments have been performed for calculating the friction coefficient. Jones [1] suggested a new Reynolds Number using friction coefficient applicable in the laminar flow by comparing results obtained from both experiments of the smooth rectangular channel flow and the circular pipe flow. Jones also suggested a method to predict the friction coefficient for the rectangular channel by using the same method as that used for the circular pipe. Garcia et al. [2] pointed out the error of friction coefficient prediction for flows with no yielding force through their relative comparison analysis among various methods for predicting non-Newtonian flows in turbulent flows. Su and Gudmundsson [3] measured the drop force through various pipe flow experiments to analyze the drop force of the horizontal well. Su and Gudmundsson calculated the friction coefficient from the drop force equation and the friction forces calculated from frictional velocity near the wall of the pipe, determined by boundary layer theory. Additionally, Su and Gudmundsson presented a method for predicting the friction coefficient through the experimental research.

Studies of numerical analysis have been undertaken by many researchers. Romeo et al. [4] suggested a new method to predict the friction coefficient accurately by iteration of the Colebrook-White equation, with parameters estimated from the nonlinear multiple methods. McKeon et al. [5] combined friction data regarding on the Reynolds Number and suggested a new friction coefficient equation for the high Reynolds Number flow through friction coefficient analysis in turbulent flow. In addition, Cheng [6] proposed the friction coefficient equation for the transition region between smooth and rough pipes in turbulent flows and modified it to apply in the open channel flow through comparisons to the experiment results performed by Nikuradse. Trinh [7] derived the Blasius empirical correlation for turbulent pipe friction factors from first principles and extended to non-Newtonian power law fluids. Calomino et al. [8] researched the experimental and numerical study of free-surface flows in a corrugated pipe.

Studies with computer methods have been developed and combined. Tonye [9] suggested a way to overcome the iterative nature of the Colebrook pipe friction factor equation by using the Visual Basic for Applications (VBA) solver options provided in Microsoft Excel^TM^. A friction factor calculator that spans the entire fluid flow regime is presented together and the results are validated by comparison to results from Moody friction factor chart. A solution method for the explicit turbulent equation of Swamee-Jain through the use of Microsoft Excel functions is also presented [9]. Padilla et al. [10] illustrate the sensitivity of the friction coefficient due to changes in flow and temperature with experiments performed in a plastic pipeline prototype. Neihguk et al. [11] conducted computationally parametric studies to show that the friction factor for perforate pipe is a linear function of the porosity.

Other papers, such as that by Liakopoulos et al. [12], studied nanochannels of various degrees of wall hydrophobicity/hydrophilicity and roughness. Ramos et al. [13] proposed the friction coefficient for the turbulent flow in Newtonian fluids based on the phenomenon of momentum exchange in the region set by the geometric mean of viscosity and length. Najafzadeh et al. [14] developed a model tree with parameters, such as diameter, mean velocity, and inner roughness, in order to accurately predict friction coefficients and improve existing models that require Reynolds number and relative roughness. Mishra et al. [15] estimated the efficiency and applicability of the existing friction factor formulas. Díaz-Damacillo, Plascencia [16] proposed a new explicit formula for estimating the friction factor using six parameters. According to Pérez-Pupo et al. [17], a review of the friction factor explicit correlations is made including 48 equations.

Although various computer programs and methods have been developed for estimating pipe friction loss, research has been found to be insufficient to calculate the exact pipe friction coefficient. For every material condition, there is friction value ranged for its own value. However, sometimes these friction values are out of date or miscalculated. Previous equations on friction coefficient has limits to certain roughness of pipe or flow. Therefore, in order to estimate the pipe friction coefficient in any type of roughness and in laminar and turbulence flow, as mentioned in this study, we would like to propose an equation using entropy for estimating friction loss.

## 2. Methodology

### 2.1. Chiu’s Velocity and Darcy–Weisbach Equation

**Figure 1:**
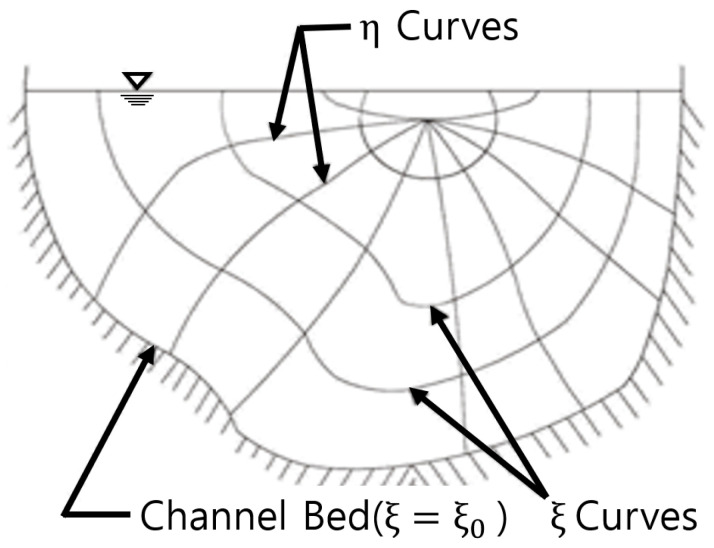
ξ−η of open channel (ξ and η are the spatial coordinates) from Chiu [18,19].

Chiu [18,19] applied the stochastic entropy equation to calculate mean velocity and suggested Equation (1), as follows,

(1) $$u=\frac{u_{\max}}{M}\ln\left[1+\left(e^{M}-1\right)\frac{\xi -\xi_{0}}{\xi_{\max}-\xi_{0}}\right]$$

where u is the velocity, $u_{\max}$ is the maximum velocity, ξ (0 ≤ ξ ≤ 1) is the spatial coordinates when the velocity is u, $\xi_{0}$ is the minimum values of ξ occurring at the channel boundary layer (u=0), $\xi_{\max}$ is the maximum values of ξ occurring at the u, M is the entropy parameter (see Figure 1).

The mean velocity equation (Equation (3)) using K(M) and water level is proposed and used, as shown in Equation (2).

(2) $$K\left(M\right)=\phi \left(M\right)\cdot M=\frac{M\left(e^{M}-e^{M}+1\right)}{\left(e^{M}-1\right)}$$

here, ϕ(M) is $\frac{e^{M}-e^{M}+1}{e^{M}-1}$

(3) $$u=\;\frac{\bar{u}}{K\left(M\right)}\ln\left[1+\left(e^{M}-1\right)\left(\frac{y-y_{0}}{y_{\max}-y_{0}}\right)\right]$$

where $\bar{u}$ is the mean velocity, y is water level for u, $y_{0}$ is the minimum water level, $y_{\max}$ is the maximum water level.

In fluid dynamics, the Darcy–Weisbach equation is used for estimating friction coefficient which is shown in Equation (4).

(4) $$h_{L}=f\cdot \frac{l}{D}\cdot \frac{v^{2}}{2g}$$

where $h_{L}$ is water head loss, f is friction loss coefficient, D is pipe diameter, v is velocity, g is gravitational acceleration.

The friction coefficient was estimated by using the equation regarding the frictional velocity, as shown in Equation (5).

(5) $$\frac{\bar{u}}{u_{*}}=\sqrt{\frac{8}{f}}$$

where, $u_{*}$ is the shear velocity.

### 2.2. Estimation of the Friction Head Loss

In this part, an equation for calculating the friction head loss using Chiu’s velocity distribution equation and friction head loss equations is purposed.

If Equation (3) is differentiated with respect to the velocity gradient, and $y_{0}=0$ and $y_{\max}-y_{0}=1$ are applied, the equation is represented as:

(6) $$\left[\frac{\mathrm{du}}{\mathrm{dy}}\right]=\frac{\bar{u}\cdot \left(e^{M}-1\right)}{R_{h}\cdot K\left(M\right)\left[1+\left(e^{M}-1\right)y\right]}$$

where $R_{h}$ is the hydraulic radius.

As for the velocity (u is zero at the boundary layer), Equation (6) can be modified as Equation (7).

(7) $${\left[\frac{\mathrm{du}}{\mathrm{dy}}\right]}_{y=y_{0}}=\frac{\bar{u}\cdot \left(e^{M}-1\right)}{R_{h}\cdot K\left(M\right)}$$

Equation (7) can be represented with shear stress equation (Equation (8)), as follows:

(8) $$\tau_{0}=\rho \nu {\left[\frac{\mathrm{du}}{\mathrm{dy}}\right]}_{y=y_{0}}$$

where $\tau_{0}$ is the bottom shear stress, ρ is the density of the fluid, ν is the kinematic viscosity:

(9) $$\tau_{0}=\rho \nu \frac{\bar{u}\cdot \left(e^{M}-1\right)}{R_{h}\cdot K\left(M\right)}$$

Additionally, Equation (9) can be represented with shear velocity equation (Equation (10)), as follows:

(10) $$u_{*}=\sqrt{\frac{\tau_{0}}{\rho }}$$

(11) $$u_{*}^{2}=\nu \frac{\bar{u}\cdot \left(e^{M}-1\right)}{R_{h}\cdot K\left(M\right)}$$

The friction coefficient equation can be determined by submitting Equation (5) into Equation (12) following as Equation (13).

(12) $$f=8\frac{u_{*}^{2}}{{\bar{u}}^{2}}$$

(13) $$f=8\frac{\nu \frac{\bar{u}\cdot \left(e^{M}-1\right)}{R_{h}\cdot K\left(M\right)}}{{\bar{u}}^{2}}$$

The proposed friction coefficient equation is as follows:

(14) $$f=\frac{8\cdot \nu \cdot F\left(M\right)}{R_{h}\cdot \bar{u}}$$

F(M) is defined as Equation (15).

(15) $$F\left(M\right)=\frac{\left(e^{M}-1\right)}{K\left(M\right)}$$

## 3. Application

In this paper, the pipe friction coefficient was proposed. The proposed equation was verified using Nikuradse’s experimental data [20]. The data were classified into two types (smooth pipe and rough pipe) and each were determined with diameter and relative roughness. Then, observed data and estimated (proposed equation) data were analyzed.

### 3.1. Smooth Pipe

The measured data are classified into five diameters of 1, 2, 3, 5, and 10 listed in Table 1, which experimental data are 125 cases in total. In the data, pipe diameter (D), hydraulic radius ($R_{h}$), shear velocity ($u_{*}$), average velocity ($\bar{u})$, maximum flow rate, kinematic viscosity (ν), Reynolds number ($R_{e}$), and friction coefficient (f) are included in Table 1.

The friction coefficient results determined from the developed equation with the data measured by Nikuradse from the smooth pipes experiments were compared. Then, entropy M values were determined for 125 cases, considering the flow rate and diameter of the smooth pipe. From these entropy M, F(M) was calculated by using Equation (14). Then, the friction coefficient was estimated from Equation (15).

**Figure 2:**
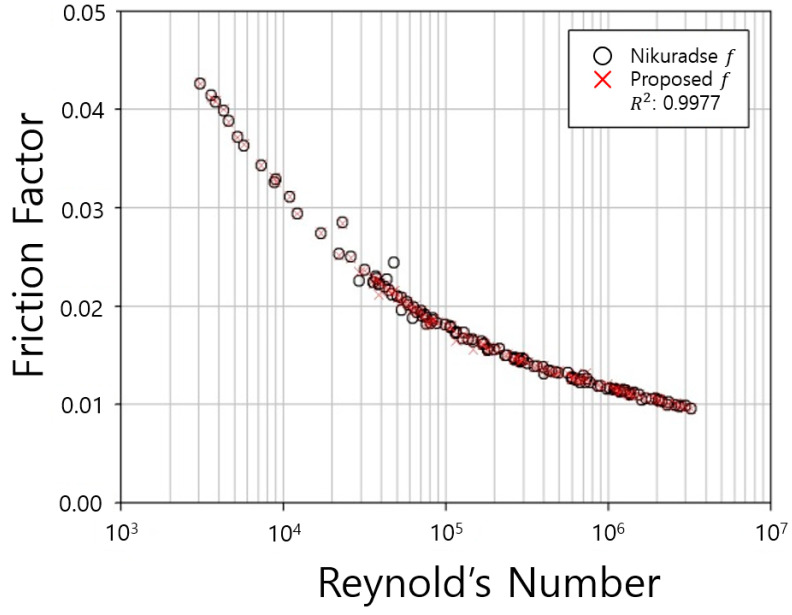
Comparison of friction factor (smooth pipe).

The estimated friction coefficients were compared with the measured data from Nikuradse’s experiments as shown in Figure 2. In Table 1, the measured friction coefficients show decrease in inverse, proportion to the diameter of the pipe. Meanwhile, the shear velocity, the average velocity, F(M) and Reynolds number rapidly increased as the diameter increased.

According to Table 1, the Reynolds numbers had quite a wide range from a minimum of 3.07 to a maximum of 3230. For the visibility of the graph, we used the logarithm scale of the axis in Figure 2. Figure 2 shows that the developed equation well represented the Nikuradse’s friction coefficients, showing very high accuracy, in which the R^2^ values was 0.9977.

### 3.2. Rough Pipe

In the case of the rough pipe experiments performed by Nikuradse, the roughness was represented with sand attached to the pipe wall. Nikuradse measured from total of 362 cases experiments and the classified into six relative roughness conditions: 507, 252, 126, 60, 30.6, 15. Each type of relative roughness was classified into three diameters of 9.94 cm, 4.94 cm and 2.474 cm. In the data, relative roughness (r/k), pipe diameter (D), hydraulic radius ($R_{h}$), shear velocity ($u_{*}$), average velocity ($\bar{u})$, maximum flow rate, kinematic viscosity (ν), Reynolds number ($R_{e}$), and friction coefficient (f) are included in Table 2.

The entropy M values were determined for 362 cases investigated by Nikuradse considering the flow rate and diameter of the smooth pipe. We also calculated F(M) using Equation (14) and determined the friction coefficient from Equation (15).

**Figure 3:**
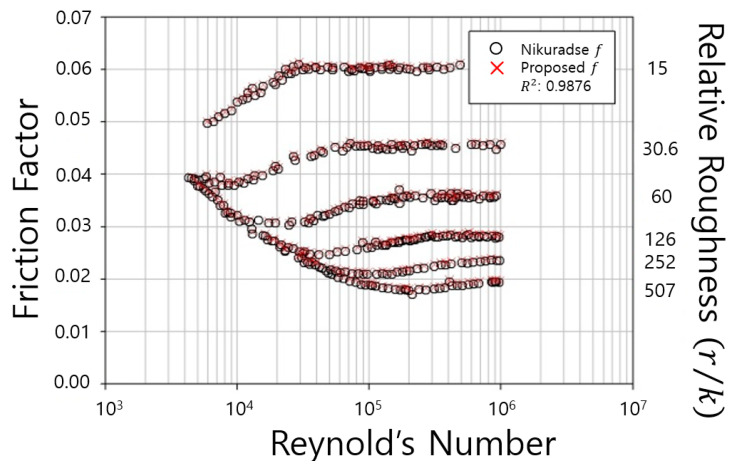
Comparison of friction factor (rough pipe).

The determined friction coefficients were compared with the measured friction coefficients from Nikuradse’s experiments as shown in Figure 3. Figure 3 show that the measured shear velocity, mean velocity and Reynolds number had a certain range, but pipe friction coefficient had a zone in narrow ranges.

For the visibility of the graph, Figure 3 also used the logarithm scale of the axis. Figure 3 shows that the developed equation well represented the Nikuradse’s friction coefficients, showing very high accuracy in which the R2 values had a range from 0.9796 to 0.9941.

### 3.3. RMSE (Root Mean Square Error)

The RMSE is a measure of the residual, which is the difference between the values predicted by the model and actual observed values. The RMSE enables predictive power to be integrated into a single unit of measurement. The RMSE of the model’s prediction for the estimated variable $X_{est,i}$ is defined as the square root of the mean square error Equation (16).

(16) $$\mathrm{RMSE}=\sqrt{\frac{\sum_{i=1}^{n}{\left(X_{obs,i}-X_{est,i}\right)}^{2}}{n}}$$

where $X_{obs,i}$ indicates the actual observed value, and $X_{est,i}$ is the predicted value obtained from the model.

## 4. Results and Discussion

**Figure 4:**
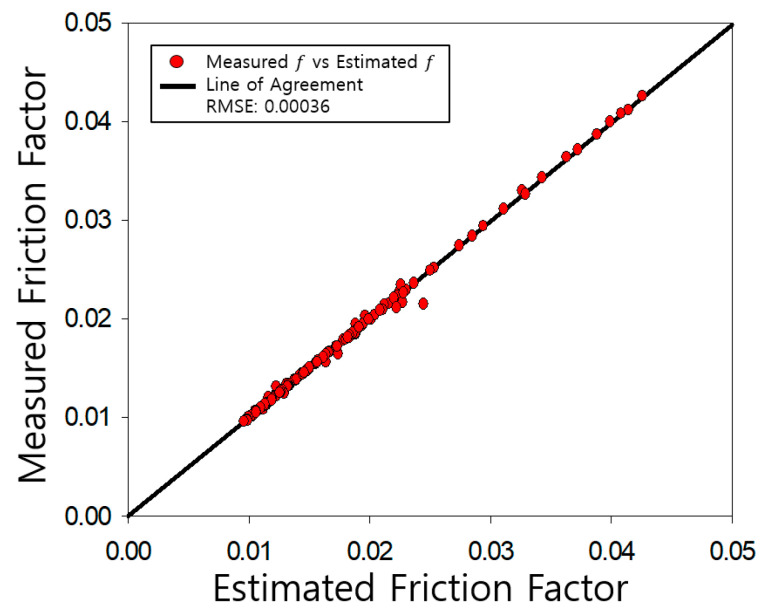
Comparison and verification of friction factor (smooth pipe).

**Figure 5:**
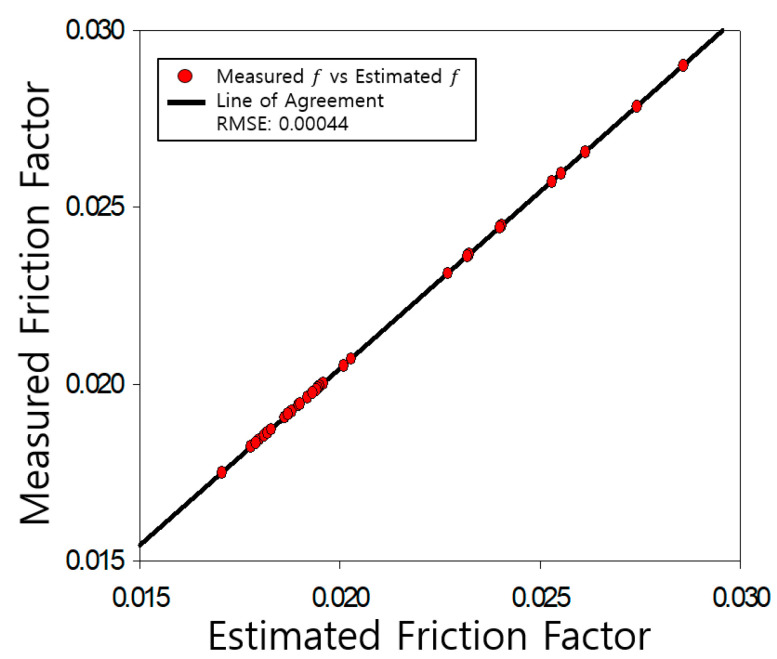
Comparison and verification of friction factor (rough pipe, r/k = 507).

**Figure 6:**
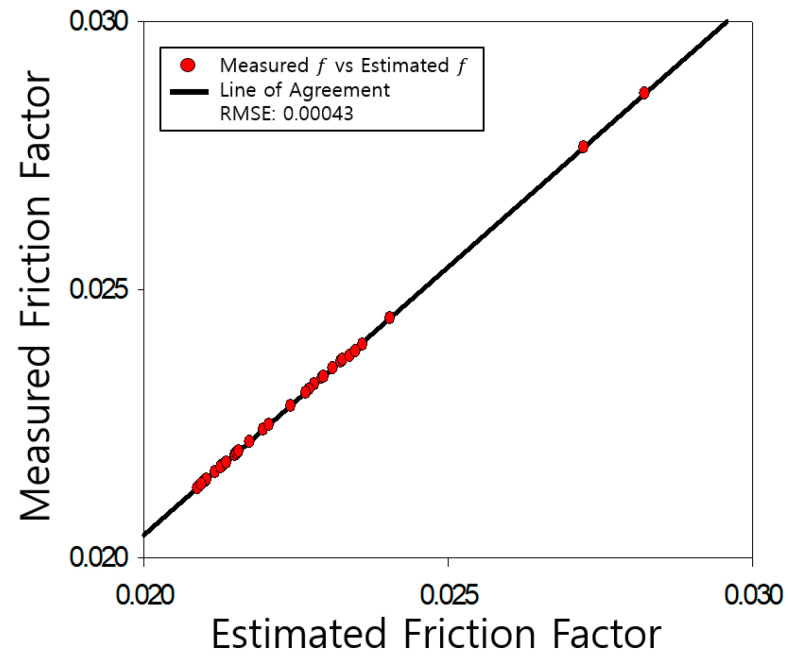
Comparison and verification of friction factor (rough pipe, r/k = 252).

**Figure 7:**
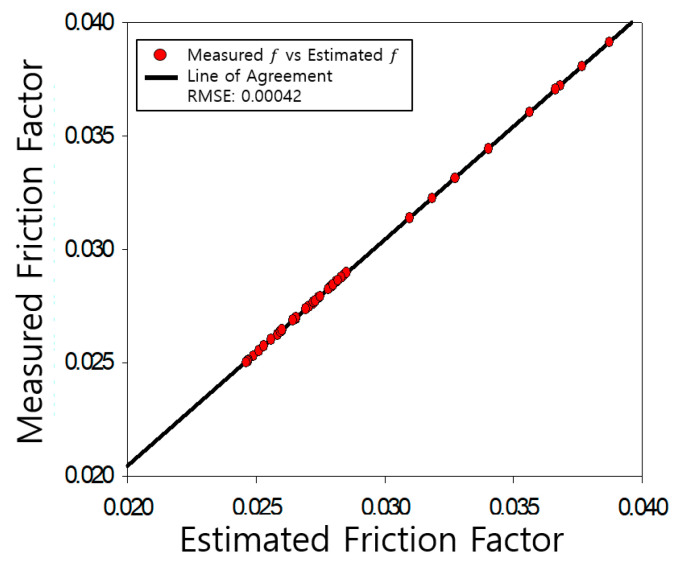
Comparison and verification of friction factor (rough pipe, r/k = 126).

**Figure 8:**
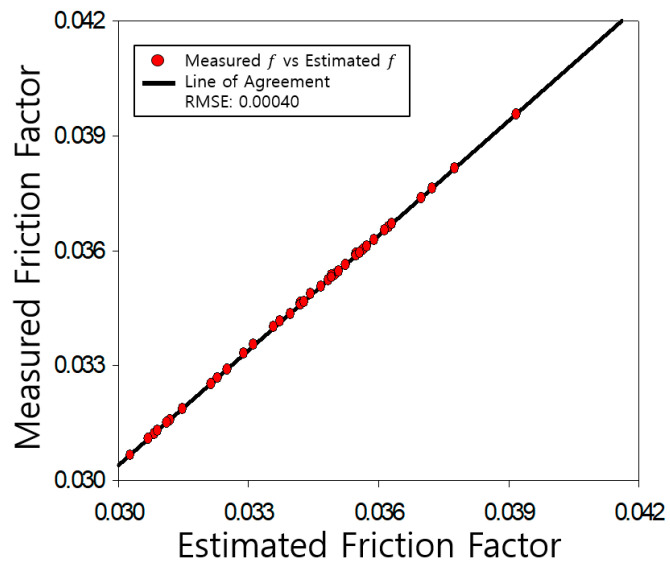
Comparison and verification of friction factor (rough pipe, r/k = 60).

**Figure 9:**
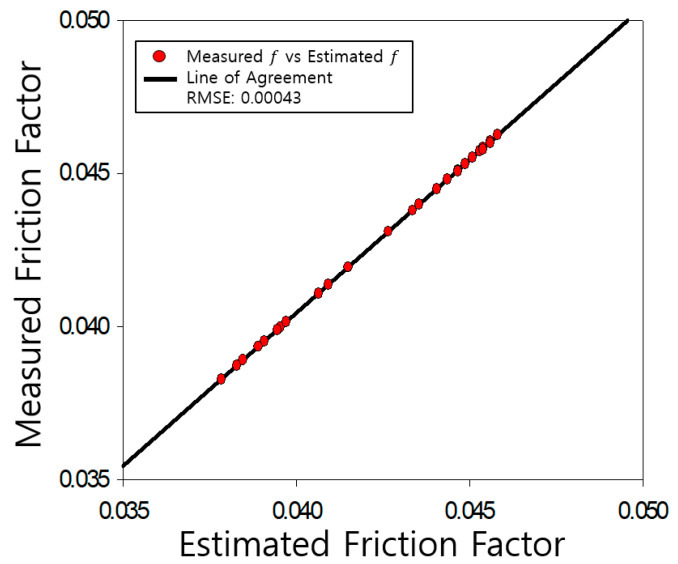
Comparison and verification of friction factor (rough pipe, r/k = 30.6).

**Figure 10:**
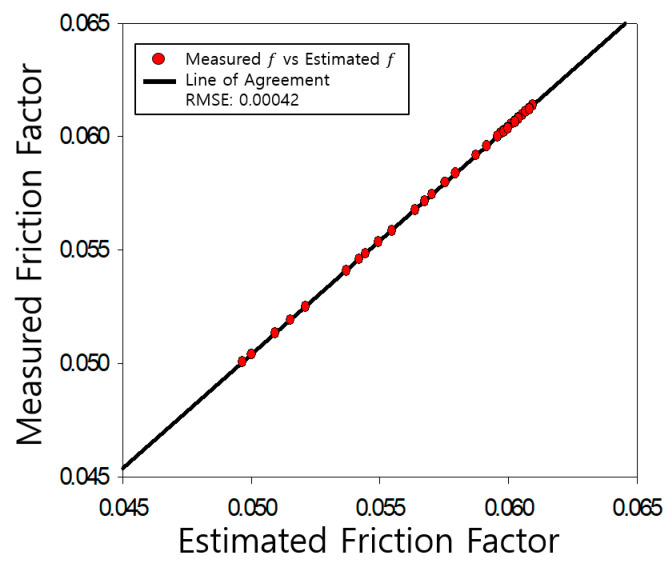
Comparison and verification of friction factor (rough pipe, r/k = 15).

The determined friction coefficients of the pipe were compared with those measured by Nikuradse, as shown Figure 4, Figure 5, Figure 6, Figure 7, Figure 8, Figure 9 and Figure 10. Each determined RMSE values were very low from 0.000366 to 0.000436 as shown in Table 3.

The more quantitative validation, this study used discrepancy ratio method which method is a statistical analysis method for calculating ratio between measured and determined coefficients Equation (17).

(17) log(estimated f/measured f)=constant

Each calculated constraint is sorted in ascending order and then expressed as a percentage of certain divided section. If the value is greater than 0, it means over-determination, and if it is less than 0, it means under-determination.

**Figure 11:**
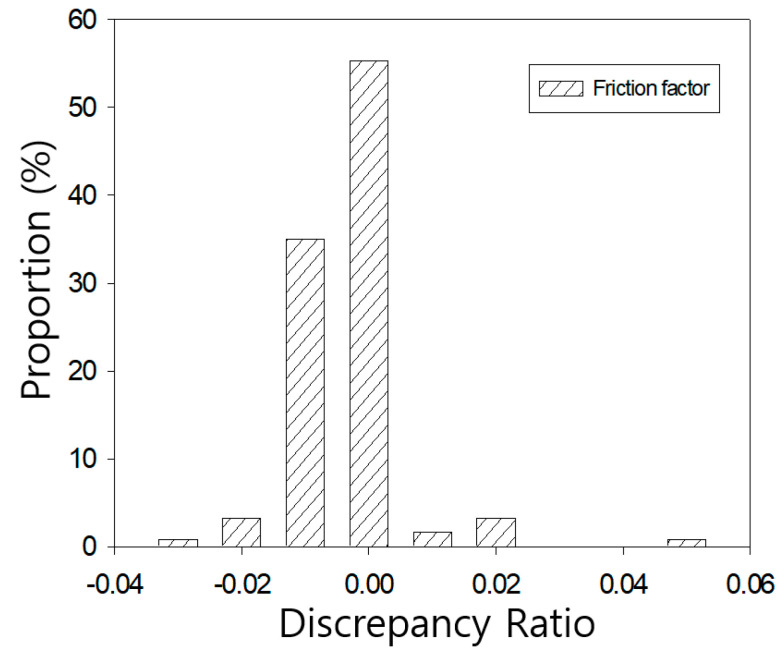
Discrepancy ratio for velocity results in smooth pipe.

**Figure 12:**
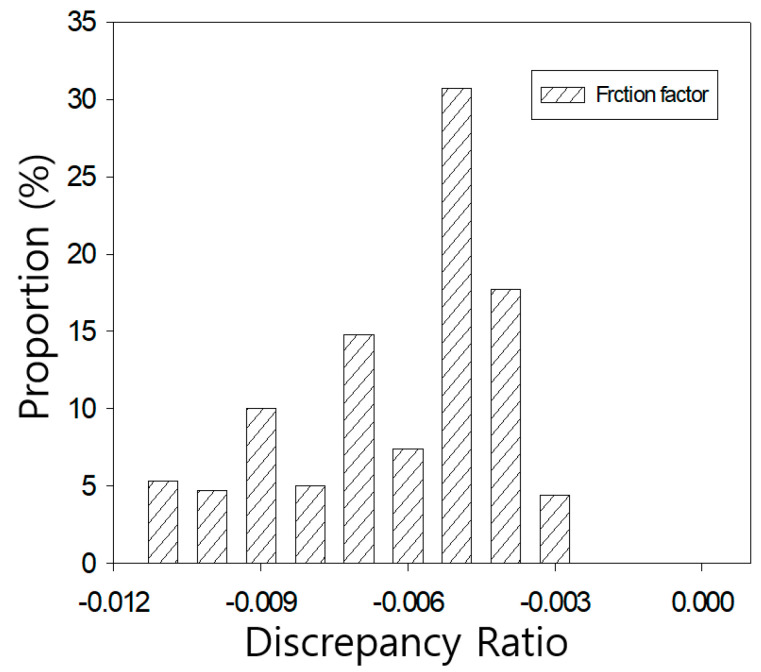
Discrepancy ratio for velocity results in rough pipe.

Determined friction coefficients were compared with Nikuradse’s results, as shown in Figure 11 and Figure 12. These figures show that the discrepancy ratio results of the proposed equation were all distributed near 0. In the case of the smooth pipe, it was found to be very close to 0 in the range of 0.055 to −0.029 as shown in Figure 11. In the case of the rough pipe, it is distributed in the range of −0.011 to −0.003 and tends to be underestimated, but the overall distribution is very close to zero as shown in Figure 12.

## 5. Conclusions

This study proposed a new equation using the entropy-based mean velocity equation for estimating the pipe friction coefficient, which is a very important factor for the determination of pipe friction loss. The proposed equation used the F(M) factor derived by combining Darcy–Weisbach’s formula for friction head loss, shear velocity equation and Chiu’s average velocity equation. Additionally, the proposed equation can be simply and easily calculated using only the pipe specifications, entropy values and average velocity without knowing the friction head loss and Reynolds number.

The evaluation results show that the proposed equation well represented the Nikuradse’s measured data and also the R^2^ value were 0.998 for smooth pipes and 0.979 to ~0.994 for rough pipes. In addition, the RMSE were determined to be 0.00036 for smooth pipes and from 0.00039 to 0.00044 for rough pipes. The discrepancy ratio had range from 0.055 to −0.029 for smooth pipes and a range from −0.011 to −0.003 in the case of rough pipes. Through these evaluation studies, the accuracy of the proposed equation was high and simple in applications. This shows that proposed equation can be applied to various pipe roughness values and laminar and turbulence flow.

In the future, it is expected that the pipe friction coefficient equation proposed in this study will enable the convenient estimation of flow rates to a greater extent than the existing equations. In addition, it will be possible to develop a new method for calculating roughness coefficients through continuous research on the proposed equation. Additionally, it will also be possible to respond to the Reynolds number through the study of the application method for F(M) used in the proposed equation.

## Figures and Tables

**Table 1:** Smooth pipe.

D	$R_{h}$	$u_{*}$	$\bar{u}$	ν	F(M)	$R_{e}\;$	f
1	0.25	3.124 ~14.206	42.8 ~311	0.014 ~0.0135	4.072 ~12.428	3.07 ~23	0.016692 ~0.042626
2	0.5	6.711 ~46.792	114.6 ~1053	0.0114 ~0.0135	14.555 ~89.625	17 ~182	0.015797 ~0.027434
3	0.75	4.895 ~46.996	91.4 ~1106	0.0114 ~0.0123	15.948 ~130.237	37 ~288	0.01444 ~0.02295
5	1.25	3.857 ~42.657	71.2 ~1082	0.0134 ~0.0081	21.517 ~259.529	29.3 ~670	0.0124344 ~0.0234805
10	2.5	11.234 ~83.999	259 ~2425	0.0122 ~0.007	112.076 ~969.883	238.8 ~3230	0.0095988 ~0.0150519

**Table 2:** Rough pipe.

r/k	D	$R_{h}$	$u_{*}$	$\bar{u}$	ν	F(M)	$R_{e}$	f
507	9.94	2.485	0.923 ~41.621	15.45 ~845	0.009 ~0.012	11.622 ~586.711	13.0017 ~970.51	0.0171 ~0.0286
252	9.94	2.485	3.753 ~47.724	72.3 ~880	0.0089 ~0.0128	37.815 ~722.652	55.9758 ~979.49	0.0209 ~0.0236
	4.94	1.235	2.578 ~59.494	43.4 ~1104	0.0086 ~0.0132	14.325 ~44.893	16.2181 ~612.35	0.0209 ~0.0282
126	9.94	2.485	7.018 ~49.213	121 ~832	0.0081 ~0.0135	85.427 ~820.413	88.1251 ~970.51	0.026 ~0.0285
	2.474	0.6185	1.597 ~43.793	22.8 ~755	0.128 ~0.133	0.524 ~12.274	2.4917 ~145.881	0.0246 ~0.0393
60	9.8	2.434	6.611 ~60.015	101 ~896	0.0092 ~0.0132	80.32 ~1027.512	74.9894 ~916.22	0.0342 ~0.037
	2.434	0.6085	1.665 ~52.158	23.8 ~795	0.0114 ~0.0128	5.54 ~182.656	4.4978 ~170.216	0.0303 ~0.0392
30.6	9.64	2.41	7.449 ~70.518	99 ~934	0.009 ~0.011	121.686 ~1425.711	85.9014 ~1000	0.0447 ~0.0458
	4.87	1.2175	5.153 ~73.023	70 ~975	0.0105 ~0.0128	36.08 ~634.153	26.6073 ~451.856	0.0426 ~0.0458
	2.434	0.6085	1.704 ~49.616	24.9 ~664	0.0107 ~0.0129	5.738 ~210.841	4.69894 ~151.008	0.0378 ~0.0453
15	4.82	1.205	6.515 ~142.177	75.5 ~1648	0.0072 ~0.0132	51.318 ~1957.896	27.5423 ~1018.59	0.0596 ~0.0608
	2.412	0.603	2.427 ~68.31	30.81 ~788	0.0098 ~0.0126	9.153 ~364.357	5.88844 ~192.752	0.0497 ~0.061

**Table 3:** Prediction results R^2^ and RMSE.

**Smooth**		**R^2^**	**RMSE**
		0.9977	0.000366
**rough**	507	0.9923	0.000436
	252	0.9796	0.000434
	126	0.9886	0.000423
	60	0.9875	0.000399
	30.6	0.9941	0.000433
	15	0.9846	0.000420
